# P-304. Vancomycin Resistant *Enterococcus faecium* Minimum Inhibitory Concentration Trends from 2019-2024: Have We Killed Daptomycin?

**DOI:** 10.1093/ofid/ofae631.507

**Published:** 2025-01-29

**Authors:** Christen J Arena, Jessica L Mulbah, Rachel M Kenney, Anita Shallal, Susan L Davis, Michael Veve

**Affiliations:** Eugene Applebaum College of Pharmacy and Health Sciences, Wayne State University and Henry Ford Health, Royal Oak, Michigan; Henry Ford Hospital, Detroit, Michigan; Henry Ford Hospital, Detroit, Michigan; Henry Ford Health, Detroit, Michigan; Wayne State University, Detroit, Michigan; Henry Ford Health, Detroit, Michigan

## Abstract

**Background:**

Vancomycin resistant *Enterococcus faecium* (VRE) has been categorized as a bacterium with serious antibiotic resistance threats in the United States, where daptomycin (DAP) has been a drug of choice. In 2019, the Clinical and Laboratory Standard Institute M100S 29^th^ edition revised DAP breakpoints for VRE to susceptible dose dependent with a minimum inhibitory concentration (MIC) of ≤ 4 mcg/mL. Higher MICs are associated with resistant mechanisms and increased microbiological failure is seen with MICs of 3-4 mcg/L. Linezolid (LZD) is an VRE treatment option that has favorable clinical outcomes and microbiological eradication with less emerging resistance, but LZD use can be controversial due to outdated dogma of using a bacteriostatic agent. The purpose of this study was to evaluate MIC trends for DAP and LZD in patients with VRE bloodstream infections (BSI) at a large academic medical center.

Bloodstream Infection VRE Comment within the Electronic Health Record: Current and Proposed

**Figure:**
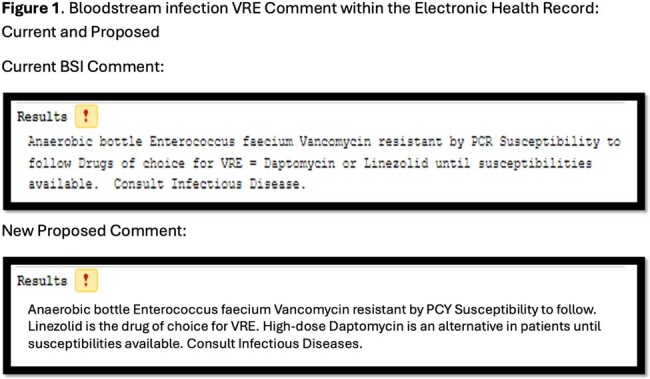

**Methods:**

IRB-exempt, cross-sectional study of adult patients with VRE BSI and a healthcare encounter from 2019-2024 at a 5-hospital health-system in southeast Michigan. Patients were identified using Microsoft SQL Server queries based on microbiology results. The primary outcome was the proportion of patients with a DAP MIC > 1 mcg/mL with corresponding LZD MICs. Our health-system currently recommends DAP or LZD for VRE BSI until susceptibilities are available via blood culture comment (**Figure 1**).

Measured MICs of daptomycin and linezolid, 2019-2024

**Figure:**
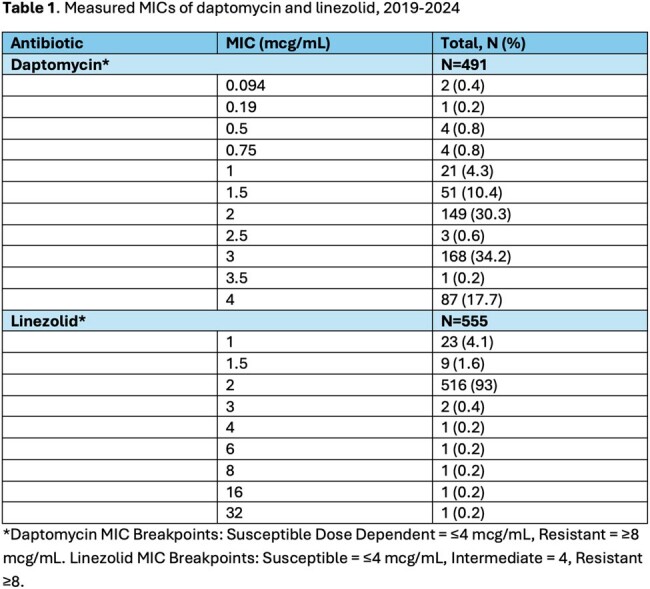

*Daptomycin MIC Breakpoints: Susceptible Dose Dependent = ≤ 4 mcg/mL, Resistant = ≥8 mcg/mL. Linezolid MIC Breakpoints: Susceptible = ≤4 mcg/mL, Intermediate = 4 mcg/L, Resistant ≥8 mcg/L

**Results:**

555 LZD and 491 DAP unique MICs from VRE BSI were evaluated. **Figure 2** represents the average MICs by month and year for DAP and LZD from 2019-2024. The majority of DAP MICs were measured at 2 (30%), 3 (56%), and 4 (18%) mcg/mL. DAP MIC_50_ and MIC_90_ values were calculated at 3 mcg/mL and 4 mcg/mL, respectively. 98.7% of LZD MICs were measured at ≤2 mcg/mL (**Table 1**). LZD MIC_50_ and MIC_90_ values were calculated at 2 mcg/mL.

Average daptomycin and linezolid MIC per month and year, 2019-2024

**Figure:**
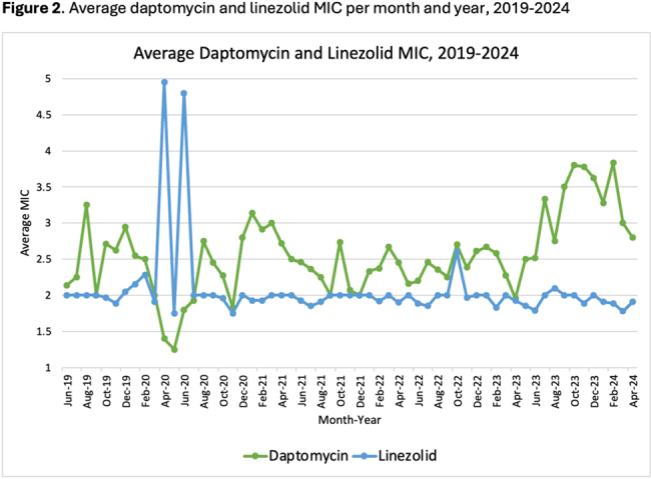

**Conclusion:**

While the VRE MICs for DAP are increasing, LZD has remained stable over the past 5 years. A possible stewardship opportunity is to modify the health-system's current blood culture comment could suggest LZD as the VRE drug of choice and high-dose DAP as an alternative in patients until susceptibilities are available (**Figure 1**).

**Disclosures:**

**Rachel M. Kenney, PharmD, BCIDP**, Medtronic Inc: Spouse is an employee, stockholder

